# Two ways of epigenetic silencing of *TFPI2* in cervical cancer

**DOI:** 10.1371/journal.pone.0234873

**Published:** 2020-06-19

**Authors:** Alexandra Fullár, Katalin Karászi, Péter Hollósi, Gábor Lendvai, Lászlóné Oláh, Andrea Reszegi, Zoltán Papp, Gábor Sobel, József Dudás, Ilona Kovalszky

**Affiliations:** 1 1^st^ Department of Pathology and Experimental Cancer Research, Semmelweis University, Budapest, Hungary; 2 Tumor Progression Research Group, Hungarian Academy of Sciences, Budapest, Hungary; 3 2^nd^ Department of Pathology, Semmelweis University, Budapest, Hungary; 4 Maternity Obstetrics and Gynecology Private Clinic, Budapest, Hungary; 5 Department of Obstetrics and Gynecology, Semmelweis University, Budapest, Hungary; 6 Department of Otorhinolaryngology, Medical University of Innsbruck, Innsbruck, Austria; Wayne State University, UNITED STATES

## Abstract

**Objective:**

Comparison of human mRNA microarray results from tumor-associated and normal cervical fibroblasts revealed significant *TFPI2* downregulation in tumor-associated fibroblasts isolated from cervical cancer, indicating that *TFPI2* downregulation may play an important role in the pathogenesis of the disease. In the present work, we investigated the mechanism of *TFPI2* downregulation in tumor-associated fibroblasts and tumor cells.

**Methods:**

*In vitro* models of monocultures and co-cultures were established with tumor cells and fibroblasts to explore the changes of TFPI-2 expression and epigenetic modifications of the *TFPI2* gene.

**Results:**

The *TFPI2* gene was hypermethylated only in tumor cells. Reduction of TFPI-2 protein levels in tumor-associated fibroblasts, although the gene was not methylated, suggested alternative regulatory mechanisms of gene expression, such as inhibition by microRNAs. The expression pattern of *miR-23a*, a gene thought to inhibit *TFPI2* translation, showed changes strongly correlated to detected TFPI-2 protein alterations. Transfections with *miR-23a* mimics resulted in a decrease of TFPI-2 protein expression whereas *miR-23a* inhibitors increased the TFPI-2 amount. Due to downregulation of *miR-23a* expression by HPV in cancer cells, *TFPI2* was silenced by promoter methylation. In contrary, *miR-23a* was active in HPV-free fibroblasts and inactivated *TFPI2*.

**Conclusion:**

These results indicate dual epigenetic inhibition of *TFPI2* on the transcription level by promoter methylation in cancer cells and on the translation level by *miR-23a* in tumor-associated fibroblasts. As a consequence, inactivation of the *TFPI2* gene plays a strategic role in the progression of cervical cancer.

## Introduction

High-risk human papilloma virus (HPV) infection is responsible for the development of cervical cancer in 99.7% of cases studied [1]. However, the majority of women infected with HPV do not develop cervical cancer, suggesting that additional environmental factors are required for tumor development.

The question addressed in this work is, whether the microenvironment could be involved in this process. Earlier we reported that the cervical connective tissue in HPV-mediated carcinogenesis plays a major role in the transformation process [2]. In the present work we demonstrate that during tumorigenesis normal stromal fibroblasts can transform into a tumor-associated phenotype. In turn, the transformed cells support the formation and subsequent invasion of cancer [3].

Cancer-associated fibroblasts (CAFs) are key players of extracellular matrix (ECM) remodeling [4]. Furthermore, by secreting matrix metalloproteinases (MMPs) and serine proteinases, CAFs are major supporters of the invasion of malignant tumors [5]. MMP-2 and MMP-9 selectively degrade the basement membrane components collagen type IV and laminin, promoting tumor cell traveling through the basement membrane. While MMP-9 is activated by MMP-2, MMP-3 and plasmin; MMP-2 is activated by MMP-14 [6, 7].

To analyze the influences of normal cervical fibroblasts and CAFs in the invasion process of cervical cancer, we compared these two cell cultures using mRNA microarray containing 41,000 target transcripts. This survey revealed that out of these targets tissue factor pathway inhibitor-2 (*TFPI2*) mRNA was the most significantly downregulated gene in CAFs.

The amino acid sequence of human TFPI-2 is identical to that of human placental protein 5 (PP5). PP5 was originally isolated from human placenta and characterized by Hans Bohn and his colleagues [8, 9]. TFPI-2 belongs to the Kunitz-type serine proteinase inhibitor (serpin) family, featuring three Kunitz-type inhibitor domains [10]. Additionally, TFPI-2 has been reported to inhibit the activation of several MMPs [11]. Ogawa *at al*. found that TFPI-2 interacts with the heparan sulfate chain of glypican-3, indicating that TFPI-2 can be tethered to the pericellular region [12]. Several recent studies verified the tumor suppressor function of TFPI-2. Indeed, its promoter is frequently silenced by hypermethylation in a series of tumors including cervical cancer [13, 14]. Furthermore, the protein can be downregulated by stably expressed *miR-616* in prostate cancer [15]. Our current report aims to elucidate the mechanism of TFPI-2 silencing in cervical cancer.

## Materials and methods

### Tissues and cell cultures

Fresh surgical specimens obtained from radical Wertheim hysterectomy from the Maternity Obstetrics and Gynecology Private Clinic and the Department of Obstetrics and Gynecology of Semmelweis University (Budapest, Hungary) were sent for routine pathology service to the 1^st^ Department of Pathology and Experimental Cancer Research of Semmelweis University. The study and the collection of surgical materials were approved by Semmelweis University Regional and Institutional Committee of Science and Research Ethics (TUKEB permit number: 95/1999). Samples were collected after written informed consent was obtained. All data were fully anonymized before authors accessed them and specimens and data were stored anonymously. Medical records of patients in S1 Table were accessed between October 2009 and September 2010. Medical records of patients in S7 Table were accessed between September 2000 and October 2019. The study conforms to the standards set by the Declaration of Helsinki.

Fibroblasts from normal (NF) and tumorous (TF, CAF) regions of uterine cervix not used for pathological diagnosis were obtained from explant cultures of the same patients. We established 3 normal and 3 tumorous primary fibroblast cultures utilized for RNA microarray. S1 Table shows the clinical characteristics of the patients. Additionally, case 2 provided a fibroblast cell culture from lymph node metastasis (MF). A pure tumor cell culture (T) has grown out from one TF culture. Ensuing experiments were carried out using these three fibroblast cell cultures (NF, MF and TF) and the tumor cell culture (T) derived from this patient.

The CSCC7 HPV16-positive cervical cancer cell line (C), derived from a squamous cervical cancer patient, was a kind gift from A. Gorter (Leiden University, Leiden, The Netherlands) [16], and used as a control tumor cell line. These cells exhibit a clear epithelial morphology and form nests when grown in monoculture. They are positive for pan-cytokeratin but negative for vimentin. In contrast, fibroblasts are vimentin positive, pan-cytokeratin negative cells displaying spindle-like morphology, with elongated, oval nuclei.

T and C tumor cells were grown as either monocultures or co-cultures with NF, TF and MF.

The raw materials and devices used in cell culture were from Sigma-Aldrich Co. (St. Louis, MO, USA) and SARSTEDT AG&Co (Nümbrecht, Germany). Reagents used in the experiments were purchased from Sigma-Aldrich Co. and Merck (Darmstadt, F. R. Germany).

### Generation of cell cultures

Tumorous and normal areas of surgical specimens were excised and cut into small pieces and placed into six-well tissue culture dishes containing AmnioGrow Plus medium (CytoGen GmbH, Sinn, Germany), optimized for development of primary cell culture. After the third passage, the growing fibroblasts were routinely transferred into DMEM-low glucose medium. Purity of the fibroblast cultures was tested by means of vimentin and cytokeratin fluorescent immunostaining. CSCC7 cells were routinely cultured in RPMI-1640 medium supplemented with 1 mM sodium pyruvate. Mycoplasma contamination was tested by PCR. All cell cultures/lines were cultured in a humidified 95% air / 5% CO_2_ incubator at 37°C and all media were supplemented with 10% fetal bovine serum (FBS), 2 mM L-glutamine, 100 unit/mL penicillin and 100 μg/mL streptomycin.

### DNA extraction and determination of HPV status

DNA was isolated using High Pure PCR Template Preparation Kit (Roche Diagnostic GmbH, Mannheim, Germany) following the manufacturer’s instructions.

Type-specific determination of HPVs was carried out by nested PCR as described previously [17]. The sequences of PCR primers are shown in S2 Table.

### Co-culture systems

Two co-culture models were used to study the interaction between fibroblasts and tumor cells. Direct co-culturing allowed physical contact between cells, whereas in indirect co-cultures cells were separated by a transwell insert with a 0.45 μm pore size allowing inter-well transport.

#### Direct co-cultures

NF, TF, MF and C cells were cultivated alone (5x10^**5**^ cells/culture) or in direct co-cultures (fibroblasts + C) at 2x5x10^**5**^ cells/culture density in a 1:1 (V/V) mixture of DMEM-low glucose and RPMI-1640. NF, TF, MF and T cells were cultivated separately (5x10^**5**^ cells/culture) or in direct co-cultures (2x5x10^**5**^ cells/culture) in DMEM-low glucose. Ninety-six hours later cell layers were collected for protein analysis in equal amount of lysis buffer. Samples from direct co-cultures are indicated as NF+T, TF+T, MF+T and NF+C, TF+C, MF+C.

#### Indirect co-cultures

Indirect co-cultures were set up for high resolution melting (HRM) methylation as follows: fibroblasts (NF, TF) were cultivated in 6-well plates (Corning Incorporated Life Sciences, Acton, MA, USA) at a density of 2.5×10^**4**^ cells/well in DMEM-low glucose. C tumor cells were placed in Transwell® polyester membrane inserts (Corning) at a density of 5×10^**4**^ cells/insert in RPMI-1640. Forty-eight hours after seeding C cells containing inserts were transferred into wells containing the fibroblast culture. This was followed by addition of 1:1 (V/V) mixture of DMEM-low glucose and RPMI-1640 to the indirect co-cultures and to the control cells growing alone. Ninety-six hours later cell layers were collected for methylation analysis. NF(+C) and TF(+C) indicate fibroblasts isolated from the bottom of the wells, whereas C(+NF) and C(+TF) designate tumor cells isolated from the bottom of the filter compartments of indirect co-culturing plates.

### DNA methylation analysis

DNA was isolated as detailed in the previous paragraph. Bisulfite conversion was performed using the EZ DNA Methylation Kit (Zymo Research, Irvine, CA, USA) according to the manufacturer’s instructions. Elution volume was 20 μL. Concentration of bisulfite converted DNA (bcDNA) samples were estimated by NanoDrop 1000 using ‘ssDNA’ settings.

Bisulfite-specific PCR (BS-PCR) reactions contained AmpliTaq Gold 360 Master Mix (Life Technologies, Carlsbad, CA, USA), LightCycler ® 480 ResoLight Dye (Roche Applied Science, Basel, Switzerland), primers at 0.2 μmol/L final concentrations, and bcDNA samples (20–40 ng bcDNA/reaction) in 15 μL final volumes. The final concentration of MgCl_2_ was 2.5 mmol/L. Real-time PCR amplification was carried out under the following conditions on a LightCycler 480 Instrument II (Roche Applied Science): denaturation at 95°C for 10 min; 10 touchdown cycles at 95°C for 30 s, 60°C (with 0.4°C decrease/cycle) for 30 s, and 72°C for 30 s; and 50 cycles of amplification at 95°C for 30 s, 56°C for 30 s, and 72°C for 30 s.

Illumina Infinium HumanMethylation450k BeadChip data available at The Cancer Genome Atlas (TCGA) database (https://cancergenome.nih.gov) were used to select CpG sites differentially methylated in cervical cancer. BS-PCR primers (S3 Table) flanking differentially methylated CpG sites were designed with the PyroMark Assay Designer software SW 2.0 (Qiagen GmbH, Hilden, Germany) to amplify bcDNA without discriminating between methylated and non-methylated sequences. Primer specificities were also verified *in silico* using the BiSearch algorithm (http://bisearch.enzim.hu).

HRM analysis began with denaturation at 95°C for 1 min, cool down to 40°C, and hold for 1 min, then continuous warm up to 95°C with 20 acquisitions/°C during melting curve fluorescence acquisition. Cp values and normalized melting curves were retrieved using the LightCycler 480 software release 1.5.0 (Roche Applied Science). For visualization of melting temperatures (T_m_), fluorescence melting peaks were obtained by plotting the negative derivative of fluorescence over temperature (-dF/dT) versus temperature (T). In order to calibrate our methylation sensitive HRM (MS-HRM) assays, *in vitro* fully methylated and unmethylated bcDNA samples (EpiTect Control DNA, Qiagen) were mixed in different DNA methylation ratios (0%, 25%, 50%, 75%, 100%) and analyzed by MS-HRM. Average methylation levels of all samples were estimated after visually monitoring the melting points by two independent observers and comparing with those of standard samples.

Pyrosequencing of BS-PCR products was performed on a PyroMark Q24 instrument (Qiagen GmbH, Hilden, Germany) using PyroMark Gold Q24 Reagents (Qiagen) and the PyroMark Assay Design software, SW 2.0 (Qiagen) according to the manufacturer’s recommendations. Sequencing results were analyzed using the PyroMark Q24 software v2.0.6 (Qiagen). Pyrosequencing results were visualized in a heat map.

### RNA extraction, human mRNA microarray, reverse transcription and real-time PCR

Total RNA was isolated using RNeasy Plus Mini Kit (Qiagen). Integrity of RNA was confirmed on Experion Automated Electrophoresis System (Bio-Rad Laboratories GmbH, Münich, Germany).

High integrity RNA samples were subjected to a human mRNA microarray with 41,000 target sequences referred to as the Whole Human Genome Oligo Microarray Kit 4x44K (Agilent Technologies, Inc., Santa Clara, CA, USA) following the manufacturer’s instructions.

Total RNA reverse transcription (RT) and real-time PCR from samples were done as indicated [18]. Primers targeting *TFPI2* (Forward: 5’-AACGCCAACAATTTCTACACCT-3’, Reverse: 5’-TACTTTTCTGTGGACCCCTCAC-3’) and GAPDH (Forward: 5’-ATCATCCCTGCCTCTACT-3’, Reverse: 5’-CTGCTTCACCACCTTCTTGA-3’) were used as reference genes. Results were obtained as threshold cycle values. Expression levels were calculated by using the 2^-ΔCT^ method.

### Immunocytochemistry and protein expression

Cells from direct co-cultures were grown on coverslips for 72 h. After fixation with ice-cold methanol (10 min) and acetone (1 min), cells were stained with H&E. Pictures were taken by Olympus BX50 (Olympus Corporation, Tokyo, Japan) microscope.

Immunofluorescent staining was performed on methanol-acetone-fixed glass plates according to standard protocols [18]. Antibodies are listed in S4 Table. Nuclei were stained with 1 μg/mL DAPI (Sigma-Aldrich, St. Louis, MO, USA). Photographs were taken with a Nikon Eclipse E600 microscope (Nikon Corporation, Tokyo, Japan) operated by the Lucia Cytogenetics version 1.5.6 program (Laboratory Imaging, Praha, Czech Republic) or with a confocal laser microscope Bio-Rad MRC 1024.

For Western blot, cells were extracted in lysis buffer (10 mM Tris-HCl pH = 7.5, 1% SDS, 95°C, 2 mM sodium orthovanadate, 10 mM sodium fluoride, 0.5% protease inhibitor cocktail; all Sigma-Aldrich). Isolated proteins were analyzed by Western blot as described previously [18, 19]. An amount of 20 μL (co-culture study) or 10 μg (miRs) of each sample was loaded per lane. Antibodies are listed in S4 Table. Western blot was normalized to known amounts of GAPDH or β-actin.

### Analysis of miRNAs targeting *TFPI2*

#### Databases

To be able to discern miRNAs targeting *TFPI2*, miRNA target prediction algorithms were used to *in silico* analysis of *TFPI2* 3′ untranslated region (UTR), which identified potential miRNA binding sites. Databases led to the identification of *miR-616*, a miRNA directly targeting *TFPI2*, validated in prostate cancer cells [15]. The miRNA search using miRNA body map (http://mellfire.ugent.be/public/body_map/) based on its several databases as TargetScan, TargetScan_cons (www.targetscan.org), Exiqon (www.exiqon.com/microrna-target-prediction), miRDB (mirdb.org/miRDB), DIANA (http://snf-515788.vm.okeanos.grnet.gr/), Pita (genie.weizmann.ac.il/pubs/mir07/mir07_dyn_data.html) and RNA22 (https://cm.jefferson.edu/data-tools-downloads/rna22-full-sets-of-predictions), resulted in a list of miRNAs from which the following five miRNAs were chosen: *miR-616-3p* (predicted by 3 databases: Exiqon, DIANA, RNA22), *miR-646* (predicted by 5 databases: TargetScan, Exiqon, miRDB, Pita, RNA22), *miR-554* and *miR-3529-5p* (predicted by 3 databases: TargetScan, Exiqon and miRDB) and *miR-23a* (predicted by 4 databases: TargetScan, DIANA, TargetScan_cons and Pita).

#### RT and qPCR

The expression of individual miRNAs was determined using TaqMan MicroRNA Assays (Life Technologies, Carlsbad, CA, USA): *miR-616* (ID: 002414), *miR-646* (ID: 001599), *miR-554* (ID: 001522), *miR-3529-5p* (ID: 463411_mat) and *miR-23a* (ID: 000399). RT and qPCR were performed according to the manufacturer’s instructions. Briefly, RT reaction was carried out using the TaqMan MicroRNA Reverse Transcription Kit in a final volume of 7.5 μL containing 10 ng total RNA. The qPCR was performed using TaqMan Universal PCR Master Mix No AmpErase UNG in a final volume of 10 μL containing 0.65 μL RT product. The amplification reaction was run in triplicates on a LightCycler 480 Instrument II (Roche Diagnostics, Indianapolis, IN). Relative expression was calculated by the 2^**-ΔΔCq**^ formula, applying *miR-195* (ID: 000494) as the most stable reference from other short RNA candidates (*miR-191* and *U6*) determined by the NormFinder application [20] and normalized to ΔCq value of normal fibroblasts.

### Transfection of *miR-23a* inhibitors and mimics

To verify the role of *miR-23a* gene, miRCURY LNA^TM^ microRNA Mimics and Power Inhibitors of *miR-23a-5p*, *miR-23a-3p* and negative controls (Exiqon, Vedbaek, Denmark) have been used to the functional analyses of normal cervical fibroblast cells (S5 and S6 Tables).

Normal cervical fibroblast cells (NF) were plated in 6-well plates, cultured with DMEM-low glucose supplemented 10% AmnioGrow Plus medium, and maintained under normal tissue culture conditions for 72 h. The medium was changed to a variant without penicillin/streptomycin (1750 μL) 24 h before transfection. MiRCURY LNA^TM^ microRNAs were transfected using Lipofectamine 3000^®^ (Thermo Fisher Scientific) according to the suppliers’ (Exiqon and Invitrogen) instructions. Briefly, to each well microRNA (2 μL of Power Inhibitors or 0.25 μL of Mimics) was mixed with 125 μL of OPTI-MEM (Thermo Fisher Scientific) in a microcentrifuge tube. In a separate tube, Lipofectamine 3000^®^ reagent was diluted in OPTI-MEM. The contents of the two tubes were then mixed in ratio 1:1 (V/V) and incubated at room temperature for 20 min. The microRNA/lipid complex mixture (250 μL) was added to the cells in culture. After 24 h, the medium was changed back to the one containing antibiotics. Seventy-two hours after transfection cells were harvested and frozen for protein analyses.

As positive control, one parallel culture remained without miR transfection (untreated-UT). After the first transfection, fluorescent-labeled negative miRs were detected in the transfected cell culture under confocal laser microscope (Bio-Rad MRC 1024) to observe the efficiency of transfection. All transfection experiments were performed in triplicates.

### Immunohistochemistry

Formalin-fixed paraffin-embedded (FFPE) cervical cancer tissue sections were stained with hematoxylin-eosin for histopathological evaluation. Approximately 30 specimens (S7 Table) not used for diagnosis, including the specimens characterized in tissue culture study, were immunostained with TFPI-2 using the Novolink Polymer Detection System (Peroxidase/DAB+, Rabbit, Novocastra Laboratories, Newcastle, UK). After inhibition of endogenous peroxidases with 10% H_2_O_2_ in methanol for 20 min, antigen retrieval was carried out by incubating slides at 100°C in TRS (10 mM Tris; 1 mM EDTA; 0.05% Tween 20; pH = 9; 3 min). Unspecific binding was blocked for 10 min using Novocastra™ Protein Block. Slides were incubated with rabbit polyclonal anti-PP5/TFPI-2 antibody (a kind gift from Dr. Hans Bohn, dilution 1:1,000) overnight at 4°C. The Novolink Polymer was applied for 30 min. The primary antibody binding to tissue sections were visualized using DAB for 10 min, and counterstained with hematoxylin. Stained slides were digitally scanned by a high-resolution bright field slide scanner (Pannoramic P1000, 3DHistech Ltd., Budapest, Hungary).

### Statistical analysis

Data were analyzed using Microsoft Excel v.2016 (Microsoft Corp., Redmond, WA, USA) and GraphPad Prism 7 (GraphPad Software, La Jolla, CA, USA). In case of mRNA array and its validation the expression data showed normal distribution and differences between NFs and MFs and between NFs and TFs were analyzed by paired Student’s t-test. Statistical significance was considered at *p* < 0.05.

## Results

### Expression of *TFPI2* precursor and mRNA in NF and TF primary cells

**Fig 1:**
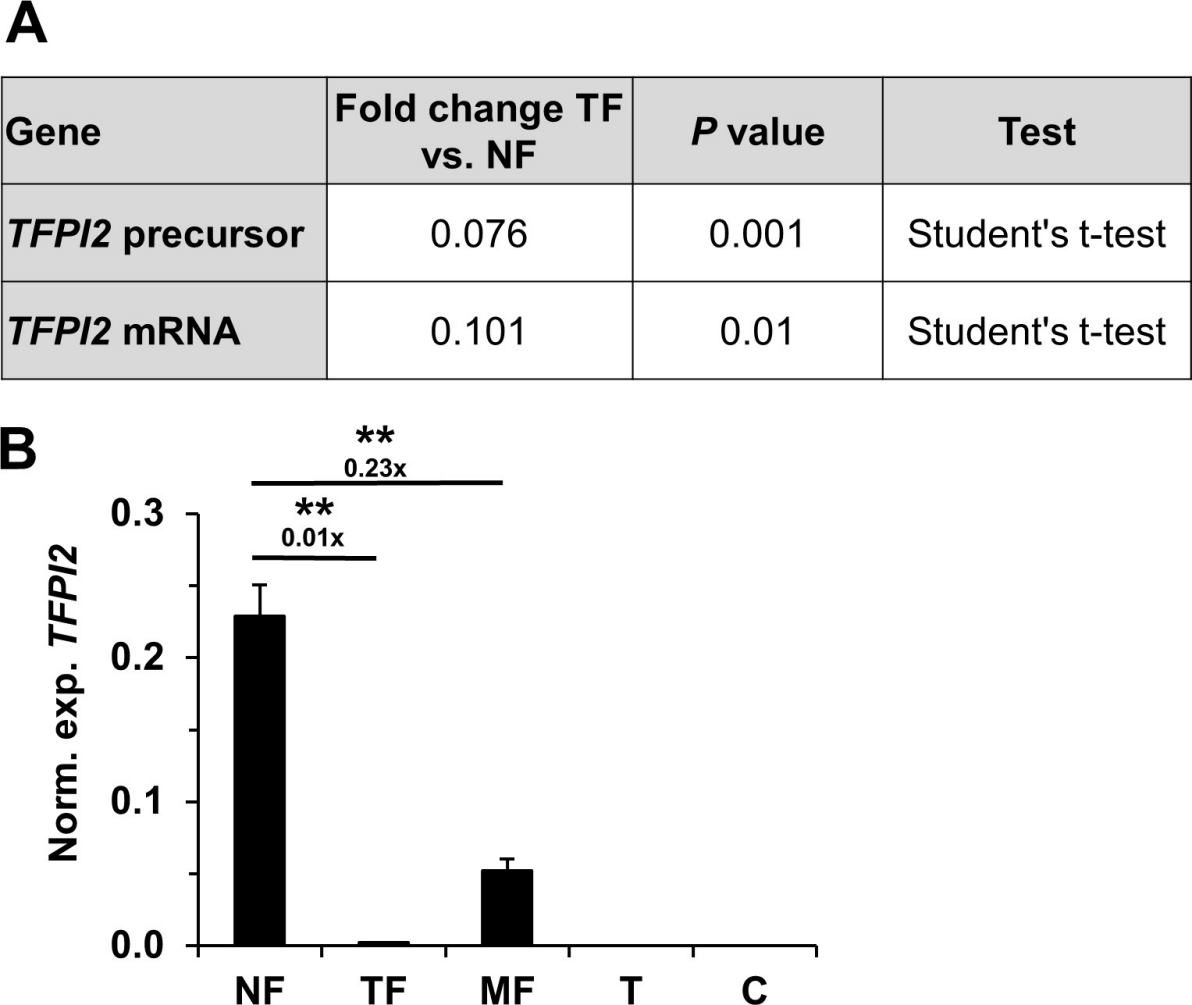
Expression of *TFPI2* in fibroblasts and cancer cells. Among 67 significantly changed mRNAs, *TFPI2* and its precursor in TF were most significantly downregulated compared to that in NF cells (panel A). The human mRNA microarray was validated by real-time PCR on cell cultures derived from explants of case 2 (S1 Table) and on C cell line. The mRNA levels of *TFPI2* changed 0.01-fold (Student’s t-test: *p* < 0.001) in TF and 0.23-fold (Student’s t-test: *p* < 0.001) in MF compared to NF. *TFPI2* was not expressed either in T or in C (panel B). Stars indicate significance: **p < 0.01. Results were expressed as mean, error bars represent SD (n = 3).

Isolated mRNA from 3 paired NF and TF samples were compared using a human mRNA microarray. Analysis revealed 67 genes (GEO: GSE148747) with significant differences among them. The highest decrease in mRNA in TF was coding for *TFPI2* (NM_006528) and its precursor molecule ([Source: Uniprot/SWISSPROT; Acc: P48307] [ENST00000222543]) compared to the *TFPI2* mRNA level of NF (Fig 1A).

The results were confirmed by real-time PCR on cell cultures derived from the explants of the second patient (S1 Table) and on C. In this measure, the *TFPI2* mRNA levels changed 0.01-fold (Student’s t-test: *p* < 0.001) in TF compared to NF. Additionally, *TFPI2* mRNA levels were decreased in MF compared to NF, although the change was less extensive (0.23-fold, Student’s t-test: *p* < 0.001) than in TF. *TFPI2* was expressed neither in T nor in C (Fig 1B).

### Characteristics of fibroblasts and tumor cells in tissue cultures

In addition to NF and TF cell cultures, we established a metastatic fibroblast (MF) cell culture derived from the lymph node of case 2 (S1 Table).

**Fig 2:**
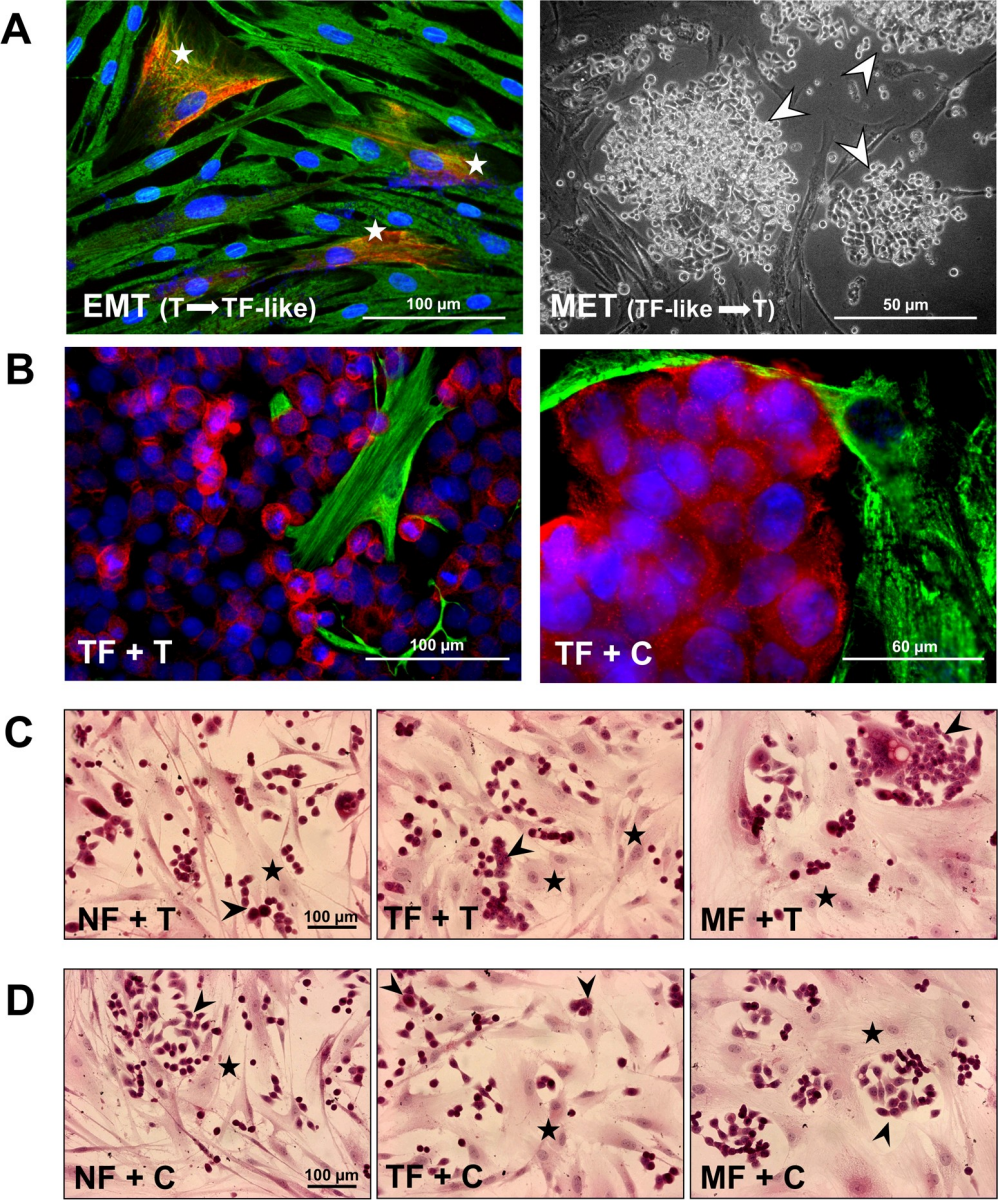
Morphology of the cells used for the experiments and typical co-cultures. In one of the tumor-associated fibroblast cultures (TF) a few vimentin and cytokeratin double positive cells were observed. These TF-like cells (marked with white star) corresponded to EMT transformed cancer cells. These newly formed cells started to proliferate and displaced the real fibroblasts from the culture. They lost their vimentin positivity and transformed into round shape cytokeratin positive T cells (marked with white arrow on MET picture). MET: mesenchymal-epithelial transition, EMT: epithelial-mesenchymal transition. The representative images show 200x magnification with scale bar of 100 μm, and 100x magnification with scale bar of 50 μm (panel A). In direct co-cultures both types of tumor cells (T and C) exhibited cytokeratin positivity, while TFs were positive for vimentin (TF+T: scale bar is 100 μm; and TF+C: scale bar is 60 μm). T represents real tumor cells similar to CSCC7 (C) cells (panel B). In the H&E stained co-cultures T and the C cells were grown with NF, TF and MF (black stars), respectively. The morphology and the arrangement of the two types of cancer cells (T and C, marked with black arrows) are comparable. They have similar epithelial morphology forming nests. All types of fibroblasts display spindle-like morphology, with oval nuclei but they have small differences: NF (normal fibroblasts) cells are thin and long; TF cells are wider with more cytoplasm, while MF (fibroblasts form lymph node metastasis) cells are the biggest cells among the fibroblasts. The representative images show 200x magnifications, scale bar of 100 μm (panels C and D). Red: cytokeratin, green: vimentin.

After 10 passages, in one of the TF cultures a few cells started to change their phenotype (Fig 2A). The cells changed their shape; in addition to cytoplasmic vimentin, they also expressed cytokeratin, and their population started to increase. After a few additional passages, these cells gained exclusively epithelial phenotype with cytokeratin positivity, vimentin negativity and physically occupied the place of fibroblasts. Hereafter these new cells were considered the own tumor cell culture of the patient (T). We compared this T cell culture with CSCC7 (C), a cell line is frequently studied in our laboratory. Fig 2B shows the presence of the epithelial marker cytokeratin (red) and the lack of the fibroblast marker vimentin in T and C tumor cells. At the same time, TFs were negative for cytokeratin and positive for vimentin (green) in both types of direct co-cultures (TF+T and TF+C).

NF, TF and MF fibroblast cells were cultured in direct co-cultures with T (Fig 2C) or C (Fig 2D). In these H&E stained co-cultures, T and C cells showed comparable morphology. NF cells seemed thin and long; TF cells were wider, while MF cells turned to be the largest cell types among fibroblasts.

### Tumor cells downregulate fibroblast TFPI-2 expression in co-culture

**Fig 3:**
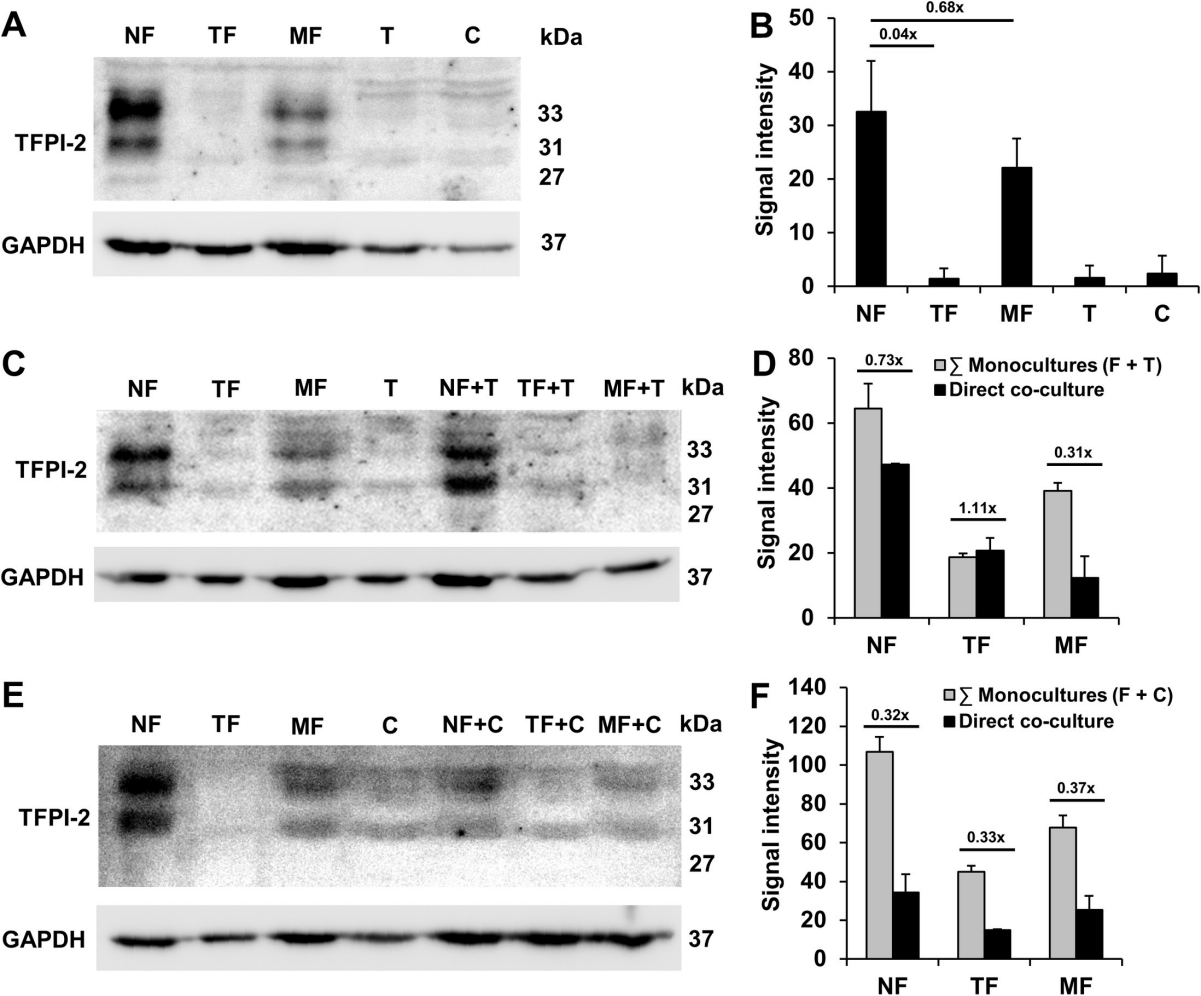
Expression of TFPI-2 protein. Western blot of TFPI-2 protein showing 33, 31, and 27 kDa bands in fibroblasts, cancer cells and their co-cultures, respectively (panels A-F). Decreasing levels of TFPI-2 protein expression was detected in fibroblasts (F) from NF, through MF to TF. Similarly to the two tumor cell cultures (T, C) the expression was hardly detectable in TF (panels A and B). Direct contact of NF or MF with tumor cells downregulated the expression of TFPI-2 (panels C-F). Thus, the final densitometric value in co-cultures was lower than the sum of densitometry in 2 monocultures (sum of monocultures F + T or F + C). ∑: sum. Results were expressed as mean, error bars represent SD (n = 2).

The expression level of TFPI-2 protein was quantified in mono- (Fig 3A and 3B) and direct co-cultures (Fig 3C–3F) using Western blot. The detected intensity values were normalized to GAPDH.

TFPI-2 protein can generally be observed in apparent molecular sizes of 33, 31, and 27 kDa [21]. In our current assay, the highest expression level was detected in NF in a monoculture model. In MF, the protein expression changed by 0.68-fold compared to TFPI-2 expression in NF. Similarly to the detected values for tumor cells (C and T), the expression was hardly detectable in TF (Fig 3A and 3B).

In direct co-culture models, the results were similar to those grown in the monocultures. The cumulative values in monocultures were higher than the measured values in co-cultures (Fig 3D and 3F) indicating a decrease of TFPI-2 expression in fibroblasts in the presence of cancer cells. In direct co-culture models, TFPI-2 protein expression was notably decreased compared to that in monoculture controls (Fig 3C–3F). Similar to the results obtained in monocultures (Fig 3A and 3B), NF exhibited the highest amount of TFPI-2 expression. A smaller amount of protein was detected in MF compared to NF, and the lowest expression level was observed in TF (Fig 3C–3F).

### TFPI-2 immunohistochemistry in human cervical cancers

The previous results indicated that TFPI-2 must be downregulated in human cervical cancer cases. Thus, immunohistochemistry was carried out on surgically removed specimens including the one we utilized for cultivation of fibroblasts, to follow the expression of the protein. Although TFPI-2 is considered a secreted protein, it could be detected on the normal cervical epithelium [22].

**Fig 4:**
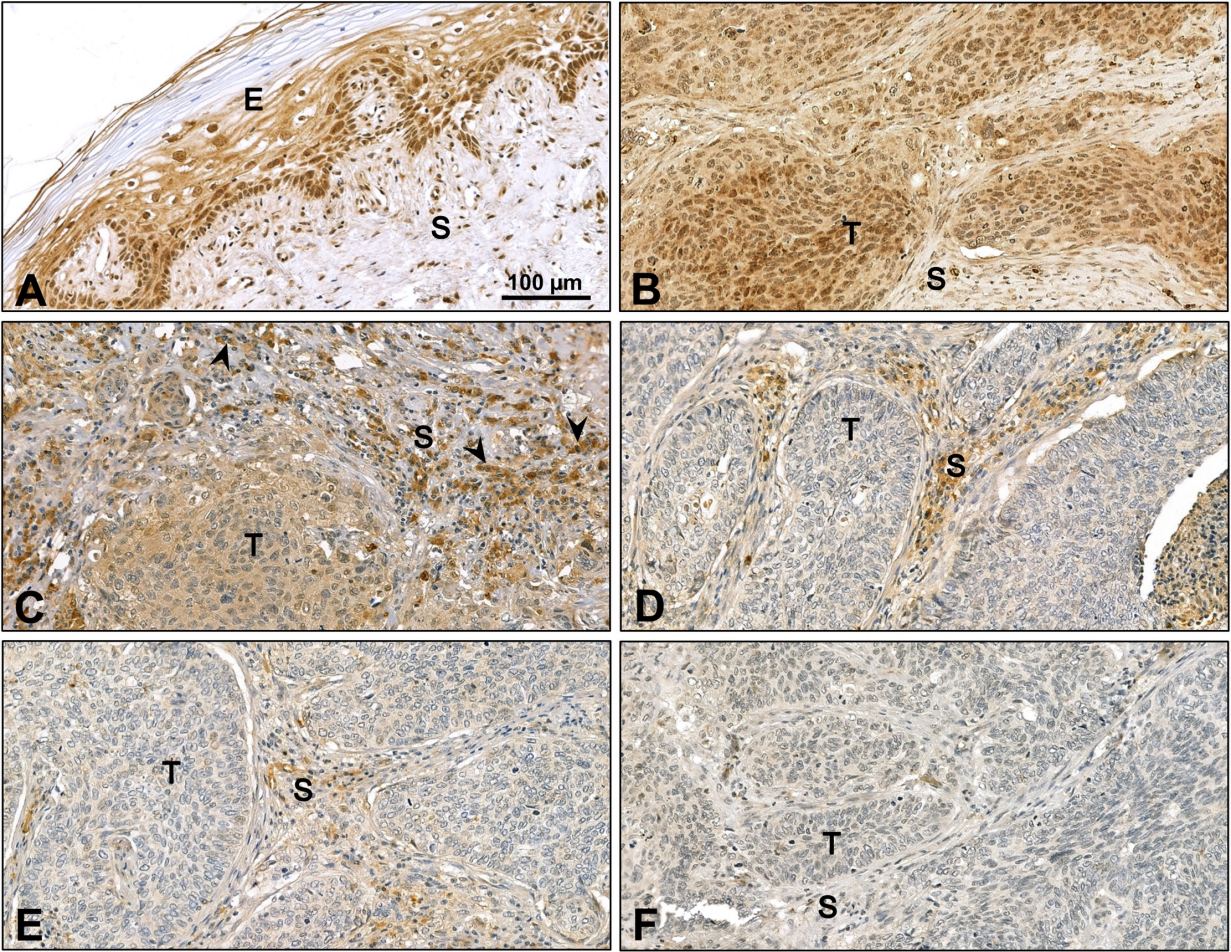
TFPI-2 immunohistochemistry of cervical cancer specimens. TFPI-2 was detected on the epithelial layer of normal cervical area. In addition to the presence in the nucleus, modest cytoplasmic positivity was observed (A). TFPI-2 was detected proximal to the cervical surface cytoplasm of tumorous nest and in lower amounts in the cytoplasm of fibroblasts (B-E). However, the deeper area of the specimens, both tumor and the surrounding connective tissue, lacked TFPI-2 staining (F). The nuclei were counterstained with hematoxylin blue. E: epithelial layer, S: stroma, T: tumor, black arrows: plasma cells. The representative images show 200x magnifications with, scale bar set to 100 μm.

While strong intra-nuclear staining was observed in the basal layer of the epithelium, the intensity was highly variable throughout the whole epithelial layer. In addition, modest amounts of TFPI-2 were detected in the cytoplasm (Fig 4A). Squamous cell cancer proximal to the cervical surface as well as in the tumor stroma expressed small amounts of cytoplasmic TFPI-2 (Fig 4B–4E). Deeper regions of the cancer specimens, both the tumorous nests and the connective tissue became negative for the protein (Fig 4F). Plasma cells in the lymph node showed strong TFPI-2 expression (Fig 4C).

### DNA methylation

**Fig 5:**
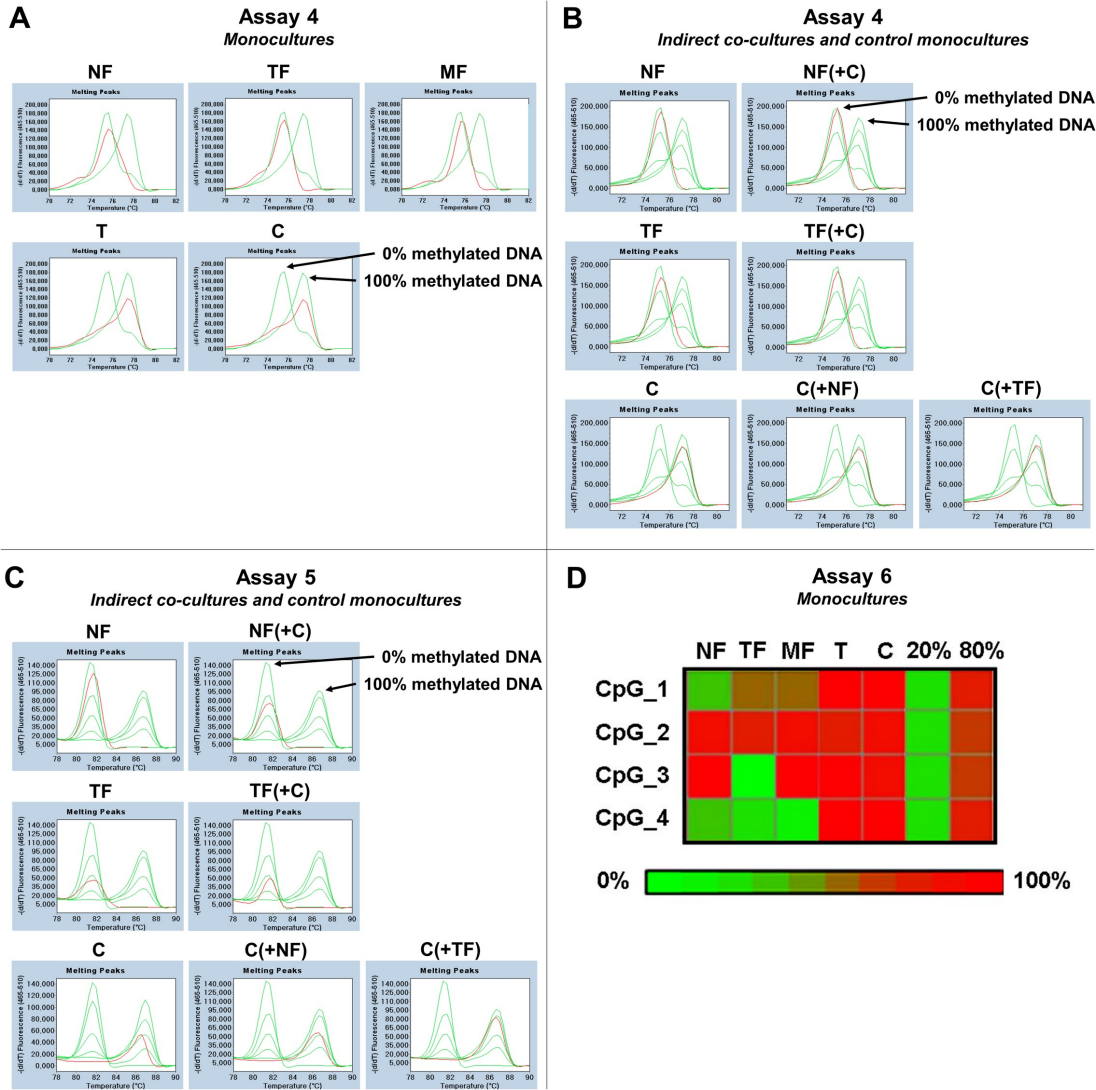
*TFPI2* gene methylation. In *assays 4* and *5* for *TFPI2* MS-HRM melting curves of cervical fibroblasts and cervical tumor cells (red line) were compared to the methylation standards (green lines with different degree of methylation: 100%, 75%, 50%, 25%, 0%) displaying characteristic melting profiles. These showed evidence of hypermethylation only in tumor cells and hypomethylation in fibroblasts both in monocultures and in indirect co-cultures (panels A-C). Differentially methylated CpG sites of *TFPI2* region in *assay 6* for cervical fibroblast and tumor cells using methylation pyrosequencing were visualized on the heat map. Both tumor cultures (T, C) showed higher methylation levels in all CpG sites, whereas the methylation level was variable in fibroblasts. Fibroblasts were not, or hardly, methylated at CpG_4 site, indicating the importance of this region in promoter silencing (panel D). Green = 100% methylated; Red = 0% methylated.

To assess the methylation status of *TFPI2* gene promoter region NF, TF and MF fibroblast cells and T and C tumor cells were analyzed by MS-HRM and pyrosequencing. MS-HRM showed methylation only in tumor cells both in monoculture and in indirect co-culture. The melting profile for these samples resembles that of the 100% methylated control, suggesting that all CpG sites within the examined amplicons were methylated in *assays 4* and *5* (Fig 5A–5C). Furthermore, pyrosequencing confirmed the methylation of *TFPI2* gene in cancer cells. The heat map shows very low levels of methylation of NF for probes CpG_1 and 4 in *assay 6* (Fig 5D). While the apparent lack of methylation of TF and MF at CpG_4 is identical with that of NF, their CpG_1 region seems slightly more extensively methylated. Based on TCGA database CpG_3 and 4 are listed as key probes to distinguish between normal and cancerous cervix (S1 Fig). The methylation pattern in our samples supports the presumption that CpG_4 is the major silencing site for *TFPI2*. The fact that CpG_3 is not methylated in TF raises doubts about the significance of CpG_3 in *assay 6*.

### *TFPI2* is silenced by microRNAs in fibroblast monocultures

**Fig 6:**
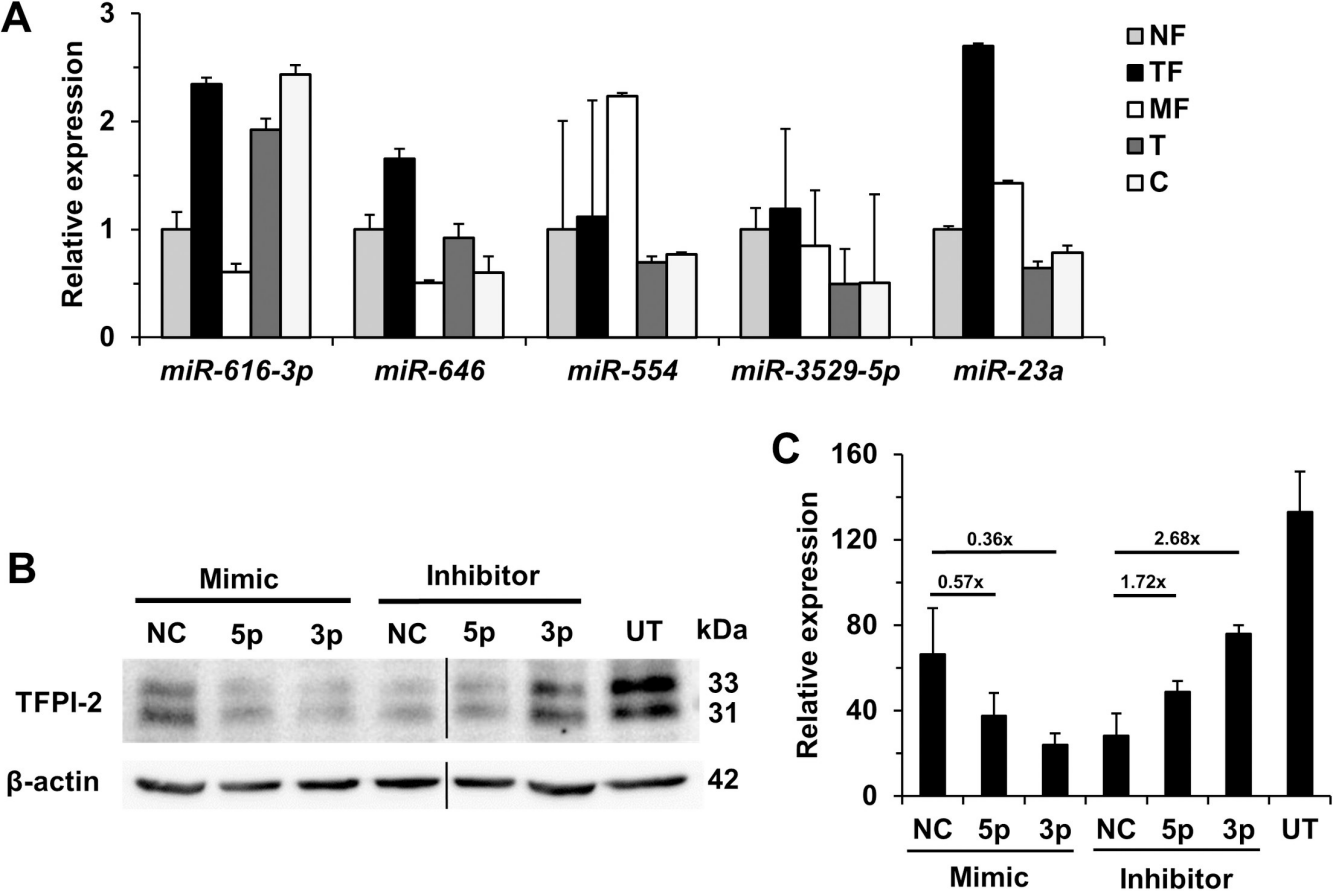
Expression of miRNAs targeting *TFPI2* in cervical cell cultures/lines. Expression of miRNAs predicted to inhibit *TFPI2* translation. Compared to NF, TF cells (black column) showed increased expression of *miR-616-3p*, *miR-646* and *miR-23a*. The changes of *miR-23a* corresponded the highest level of TFPI-2 alteration in various cell cultures (n = 3) (panel A). For validation the role of *miR-23a*, its mimics and inhibitors were transfected into NF cells. Comparing to their negative control (NC) the mimics decreased and the inhibitors increased the expression of TFPI-2 supporting the significance of *miR-23a* in the regulation of the protein. Black line: pictures were edited to remove one irrelevant lane (panel B). Densitometry indicated that *miR-23a-3p* affects mostly TFPI-2 protein expression. UT: untreated NF used as positive control (n = 2) (panel C). Results were expressed as mean, error bars represent SD.

To shed light on the key mechanism of silencing of *TFPI2* in fibroblasts (MF and TF), expression of several predicted miRNAs were tested on our cells. TF showed high expression of *miR-616-3p* and *miR-646*, but their expression pattern did not correspond to that of *TFPI2*. Namely, *miR-616-3p* expression was high in the tumor cells, where the promoter region of *TFPI2* was extensively methylated. The *miR-646* expression was low in case of MF, which raises doubt for its inhibitory action (Fig 6A). The expression profile of *miR-23a* moved the opposite direction relative to the alterations detected in TFPI-2 expression. It was high in tumor-associated fibroblasts or metastatic fibroblasts and relatively low in normal fibroblasts, and decreased in tumor cells where *TFPI2* was silenced by methylation. The latter can be related the presence of HPV E6/E7 proteins. This alteration profile indicated that *miR-23a* might be the miRNA involved in the regulation of tumor-associated fibroblasts.

### Expression of TFPI-2 protein after transfection of *miR-23a* inhibitors and mimics

Our previous results indicated that the lowest protein level of TFPI-2 was accompanied by the highest expression of *miR-23a* supporting the predictions found in 4 miRNA databases. Thus, we sought for further support to prove the role of *miR-23a* in TFPI-2 regulation. For this purpose, we selected normal fibroblasts because they have high TFPI-2 protein expression allowing the alterations easily detectable on Western blot. Untreated normal fibroblasts (UT) were used as positive control for TFPI-2 expression. Mimics and inhibitors of *miR-23a-3p*, -*5p* and their negative controls (NC) were transfected into the cells. The miR NCs reduced TFPI-2 expression. On one hand, transfection with mimics of *miR-23a* decreased the TFPI-2 protein expression in NF compared to NC of mimics. On the other, transfection with inhibitors of *miR-23a* increased TFPI-2 protein expression in NF compared to inhibitor NC (Fig 6B and 6C). As *miR-23a-3p* influenced mostly TFPI-2 protein expression in normal fibroblast cells, these results indicated that *miR-23a-3p* is a possible key factor in the inhibition of *TFPI2* translation in tumor-associated fibroblasts.

### Detection of high risk HPV in cervical cancer

**Fig 7:**
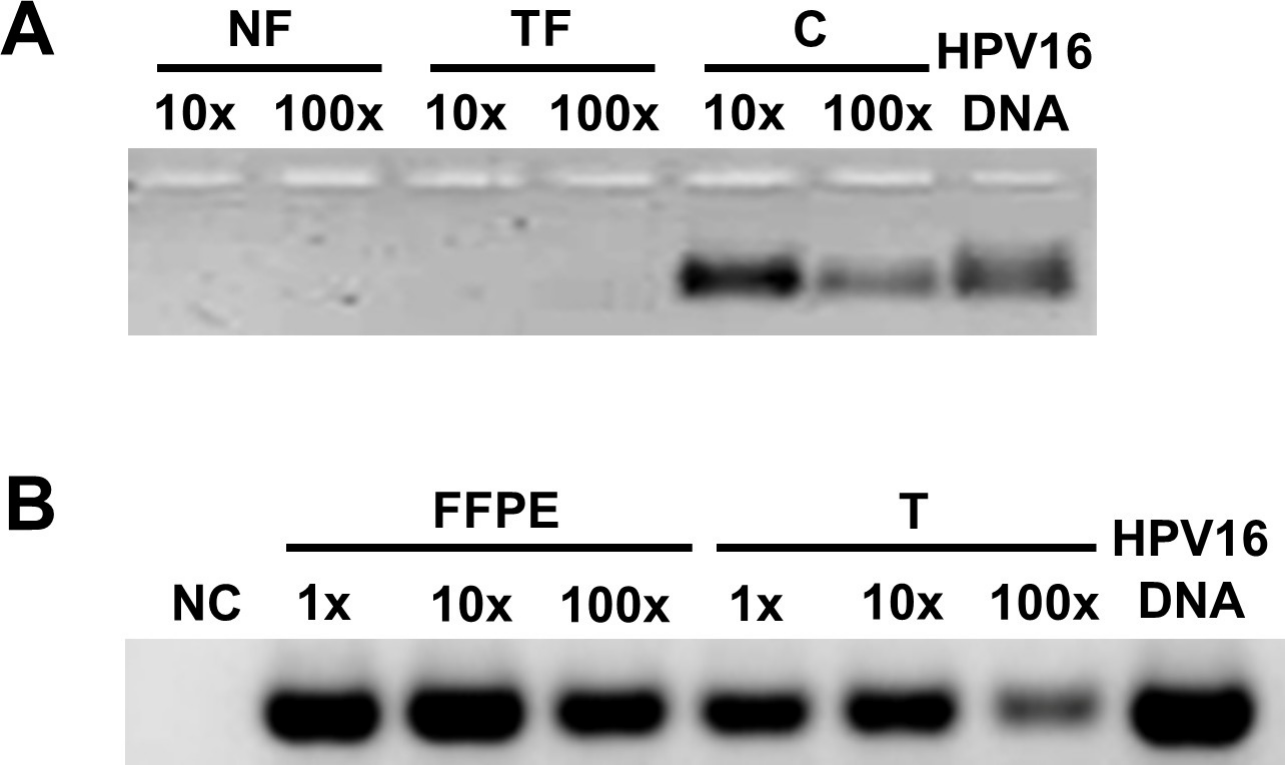
HPV status determination by HPV E6 ORF-specific nested PCR on DNAs isolated from cervical cancers. Fibroblasts (NF, TF) are HPV-free cells, and the HPV16 positivity of C tumor cells was confirmed (panel A). FFPE sample of the second case and its tumor cell culture (T) showed the presence of HPV16 (panel B). The size of PCR fragments corresponds to that of the size as the positive control. NC: negative control of first (no DNA) and second round of nested PCR; HPV16 DNA: cloned plasmid containing HPV16 DNA (positive control). The values 1x, 10x and 100x represent different dilutions.

We investigated the presence of HPV E6 DNA in fibroblasts (NF, TF), C tumor cells, the examined tumor FFPE samples (case 2) and its established tumor cell culture (T). While HPV16 E6 was clearly detectable in tumor cells (C, T) and tumor tissue (FFPE), fibroblasts remained HPV-free (Fig 7A and 7B).

## Discussion

The tumor microenvironment consists of non-tumorous cells and extracellular matrix both defining the behavior of cancer [23]. Previously we showed that CSCC7 cervical tumor cells grow significantly faster in the presence of normal fibroblasts in direct co-culture than alone indicating the supportive role of the latter in tumor growth [2]. As epigenetic changes are also critical for tumor development and progression [24, 25], we investigated epigenetic factors in both cervical tumor and its microenvironment.

Analysis of human mRNA microarray brought the *TFPI2* gene into the focus of our ensuing investigations. From 41,000 transcripts, the mRNA levels of *TFPI2* and its precursor decreased the most significantly in TFs compared to NFs. To characterize the involvement of TFPI-2 in cervical cancer, we established three fibroblast cultures: NF, MF and TF. Additionally, EMT transformed tumor cells, going through redifferentiation, forced tumor-associated fibroblasts out after a few passages (T). Establishment of the comprehensive panel of NF, MF, TF and T cell cultures allowed the detailed study of the role of TFPI-2 in the behavior of cervical cancer. Based on HPV expression status, our tumor cell culture (T) was identified as HPV16 positive, which is in a good agreement with the already established HPV16+ control CSCC7 (C) cell line [16] whereas none of the studied fibroblast cells contained the virus.

Although ample amounts of *TFPI2* mRNA and the translated protein were detected in NF, the gene expression decreased in MF and it was hardly detectable in TF, T and C monocultures. This indicated that, in addition to that of their own protein, cancer cells are capable to downregulate TFPI-2 production in tumor and metastasis-associated fibroblasts. Furthermore, apparently downregulation of the protein level is required for the progression of cancer. This presumption was supported by the immunohistochemical analysis of cervical cancer specimens where the expression of TFPI-2 gradually decreased toward to invasion front both in cancer cells and CAFs [26]. The unusual nuclear localization of TFPI-2 also have been described in mammary cancer, and it was suggested that it is responsible for modulation of the expression of MMP-2 by interacting with the Ap2-α transcription factor [27].

According to genome-wide screening data, *TFPI2* is assumed to be a tumor-suppressor gene inactivated by aberrant promoter methylation in different cancer types [13, 14]. Furthermore, compared to *HSIL*s, *CCNA1* and *TFPI2* are the most prominently methylated genes in cervical cancers. Moreover, tumors methylated for *TFPI2* are often represent a more advanced stage in cancer development [28]. Demethylation of this gene by TET2 in cervical cancer cell lines decreases cell growth, viability, and colony formation [29]. In the present work, we performed three TFPI-2 methylation assays for both cervical fibroblast and cancer cells. In agreement with the findings of Dong *et al*., in our two cervical cancer cell cultures the *TFPI2* gene promoter was methylated in both monoculture and in indirect co-culture [14]. As a consequence of promoter methylation, neither the *TFPI2* mRNA nor the protein could be detected in T and C. Surprisingly, in spite of the fact that cancer-associated fibroblasts also failed to produce TFPI-2 protein, they remained unmethylated. It seems that, cancer cell DNA methylation alone is not sufficient to inhibit TFPI-2 as fibroblasts from the cervix are capable to secrete this factor into the tumor stroma. Thus, we suggest that inhibition of TFPI-2 production in TFs further increases the aggressivity of cancer.

Based on these observations, we hypothesized that silencing of *TFPI2* in fibroblasts is an alternative process and is brought upon by inhibitory microRNAs rather than methylation. Although several studies focused on cancer cell-derived microRNAs [15], only a few explored the regulatory role of stromal cell-derived microRNAs during carcinogenesis. The specific microRNAs and their reported role in the tumor microenvironment are summarized by Soon and Kiaris [30]. Findings of Mitra *et al*. in ovarian cancer also supported the idea that fibroblast remodeling takes place through the actions of miRNAs (*miR-31*, *miR-214*, and *miR-155*) [31]. Furthermore, Kunita *et al*. demonstrated that *miR-21* expression in lung fibroblasts might trigger normal fibroblasts to transform into CAFs further supporting cancer progression [32].

Five miRNAs (*miR-616*, *miR-646*, *miR-554*, *miR-3529-5p* and *miR-23a*) were predicted *in silico* to inhibit *TFPI2* translation in tumor-associated fibroblast based on different miR databases. Their expression was determined in NF, MF, TF, T and C. Although similar to the events in prostate cancer [15] *miR-616* was overexpressed in both cancer cell cultures and in addition in TF, but its level was low in MF. Based on its expression pattern, *miR-23a* showed the expected changes that corresponded the alteration of TFPI-2. As normal fibroblasts of uterine cervix produced ample amount of the TFPI-2 protein, these cells were used for validation the role of *miR-23a*. Application of a *miR-23a* mimic downregulated, whereas *miR-23a* inhibitor upregulated TFPI-2 protein expression confirming the potential role of *miR-23a* in *TFPI2* translation regulation.

**Fig 8:**
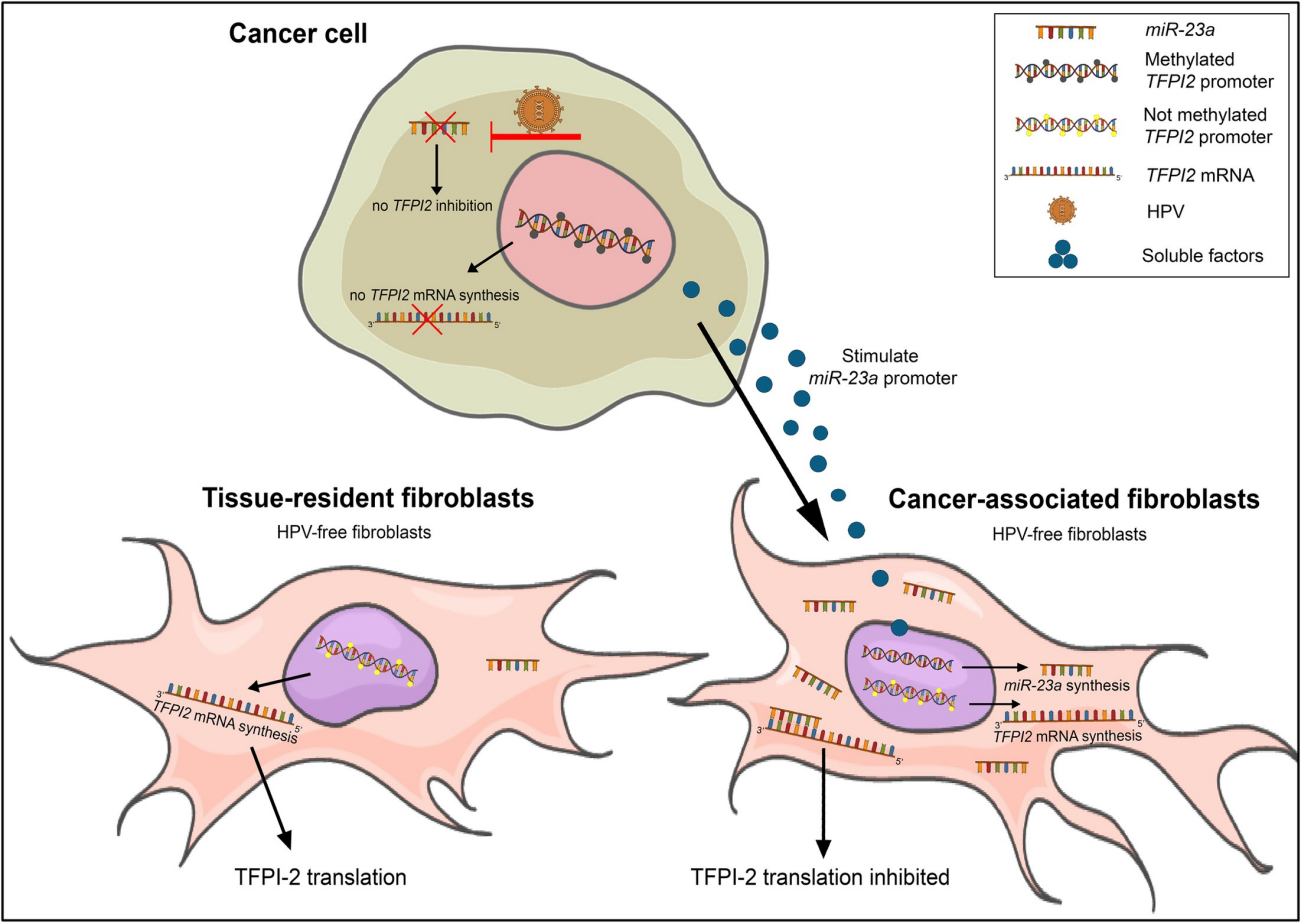
Two ways of epigenetic silencing of *TFPI2* in cervical cancer. Continuous E6/E7 expression of HPV hinders *miR-23a* in cancer cells, hereby the TFPI-2 could be expressed. However, this is overridden by promoter methylation epigenetic silencing thus finally TFPI-2 expression is inhibited in cancer cells. In spite of that *TFPI2* promoter was not methylated in fibroblasts, TFs did not produce the protein. This is explained by the elevated intracellular concentrations of *miR-23a* stimulated by a so far unknown factor(s) of cancer cells. Translation of *TFPI2* is inhibited by binding of *miR-23a* to the 3’ UTR of *TFPI2* mRNA.

The next question is, why two different epigenetic processes are needed for *TFPI2* inactivation in cervical cancer. Silencing of the E6/E7 oncoprotein in HPV virus induces a significant level of *miR-23a-3p* upregulation. This implies that the E6/E7 oncoproteins hinder the expression of *miR-23a-3p*, which, as a consequence, cannot exert its TFPI-2 inhibitory effect in HPV infected cells [33] but is capable of downregulating TFPI-2 in virus-free tumor-associated fibroblasts. To compensate for the lack of *miR-23a*, tumor cells inactivated the transcription of TFPI-2 by promoter hypermethylation (Fig 8). Although *miR-27a* upregulation also was detected in cervical cancer, its presence is in agreement with our results as *miR-23a*, *miR-27a and miR-24-2* can act in cluster [34, 35].

Although the importance of TFPI-2 has not generally been appreciated, the work of Rots and Huisman [29] elegantly highlights the significance of this protein. Nevertheless, a better understanding of the complex relationship between the two epigenetic regulatory processes of the related genes is required to uncover the intimate details of this cooperative silencing of TFPI-2.

Chou and Werb suggest that there are many unclarified questions lingering about the mechanism of microRNA action within the tumor microenvironment. For example, at this point we do not know how cancer cells initiate downregulation of *TFPI2* by miRNAs in CAFs, or what kind of promoters or other upstream signals are responsible for the upregulation of *miR-23a* [36]. We presume that a so far unknown factor(s) of cancer cells could participate in *miR-23a* upregulation in HPV-free fibroblast (Fig 8).

A recent review concluded that *miR-23a* is one of the most frequently studied miRNA implicated in the initiation, progression and management of various cancers. Furthermore, it has the potential to regulate the tumor microenvironment [37]. This observation supports our finding that TFPI-2 is downregulated by *miR-23a* in tumor-associated fibroblasts. The next step is to find out how exactly tumor cells and fibroblasts communicate.

The c-Myc suppression of *miR-23a/b* leads to the enhancement of glutaminase-regulating glutamine metabolism, a process that is paramount for the bioenergetics and maintaining the redox homeostasis in cancer cells [38].

Further research will focus on the understanding of the transcriptional regulation of *miR-23a* [37]. In addition to the factors mentioned above, AP1, SP1, CREB, p65 nuclear factor, p53, MAPK ERK5, GAS5 are all suggested as potential regulators of *mir-23a* transcription, and in the pathogenesis of cancer [38–43].

## Conclusion

In summary, we demonstrated that inactivation of the *TFPI2* gene occurs via cooperative action of cervical cancer cells and tumor-associated cervical fibroblasts, utilizing two well-known epigenetic regulatory mechanisms. Based on its strategic role in inhibition of tumor cell invasion, TFPI-2 can be considered a tumor suppressor in this type of malignant tumor.

## Supporting information

S1 Fig The 5’ untranslated region of *TFPI2* gene showing the area studied for methylation. University of California, Santa Cruz (UCSC) Genome Browser showing *TFPI2* gene region: chr7:93,885,397–93,890,991 (5,595 bp) (Human Genome Assembly GRCh38/hg38), CpG island of this region: chr7:93,890,055–93,890,872 (818 bp). TFPI2 assays were designed using PyroMark Assay Design 2.0: *TFPI2 assay 4*: chr7:93,520,202–93,520,404 (203 bp); *TFPI2 assay 5*: chr7:93,519,893–93,520,216 (324 bp) and *TFPI2 assay 6*: chr7:93,520,388–93,520,601 (214 bp). Based on TCGA database 2 probes of *TFPI2 assay 6* provides larger difference between normal and cancerous cervix: Probe cg09558850 (CpG_3 in our study): chr7:93,520,445–93,520,445 and Probe cg19854521 (CpG_4 in our study): chr7:93,520,452–93,520,452.
(TIF)

S1 Table Clinico-pathological data of the patients whose cervical explants were investigated. (DOCX)

S2 Table HPV type-specific nested PCR primer sequences. (DOCX)

S3 Table Primer sequences to *TFPI2* HRM/pyrosequencing. (DOCX)

S4 Table Antibodies used. (DOCX)

S5 Table Data of *miR-23a* mimics and inhibitors from Exiqon. (DOCX)

S6 Table Data of miRNA mimic and inhibitor negative controls from Exiqon. (DOCX)

S7 Table Clinico-pathological data of patients whose FFPE samples were used for IHC. (DOCX)

S1 Raw Images (PDF)
