# Supplementary figures and images for: Two ways of epigenetic silencing of TFPI2 in cervical cancer
Source: PLoS One. 2020 Jun 19;15(6):e0234873. doi: 10.1371/journal.pone.0234873 (PMC7304613; doi:10.1371/journal.pone.0234873)

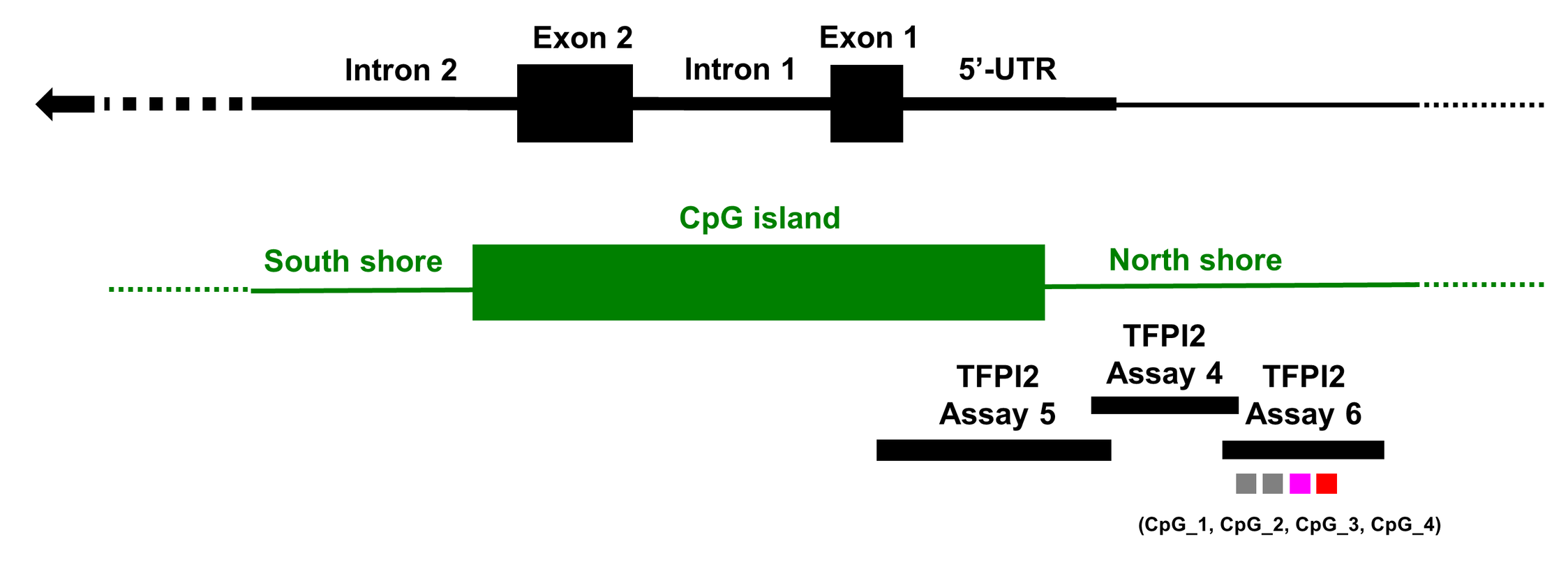

Supplement: S1 Fig — University of California, Santa Cruz (UCSC) Genome Browser showing TFPI2 gene region: chr7:93,885,397–93,890,991 (5,595 bp) (Human Genome Assembly GRCh38/hg38), CpG island of this region: chr7:93,890,055–93,890,872 (818 bp). TFPI2 assays were designed using PyroMark Assay Design 2.0: TFPI2 assay 4: chr7:93,520,202–93,520,404 (203 bp); TFPI2 assay 5: chr7:93,519,893–93,520,216 (324 bp) and TFPI2 assay 6: chr7:93,520,388–93,520,601 (214 bp). Based on TCGA database 2 probes of TFPI2 assay 6 provides larger difference between normal and cancerous cervix: Probe cg09558850 (CpG_3 in our study): chr7:93,520,445–93,520,445 and Probe cg19854521 (CpG_4 in our study): chr7:93,520,452–93,520,452. (TIF) [file pone.0234873.s001.tif]
