# Supplementary material for: Two ways of epigenetic silencing of TFPI2 in cervical cancer
Source: PLoS One. 2020 Jun 19;15(6):e0234873. doi: 10.1371/journal.pone.0234873 (PMC7304613; doi:10.1371/journal.pone.0234873)
Supplement: S1 Table — (DOCX) [file pone.0234873.s002.docx]

**S1 Table. Clinico-pathological data of the patients whose cervical explants were investigated.**

| **Case** | **Age** | **Gr** | **F** | **T** | **N** | **M** | **HPV** | **Diagnosis** |
| --- | --- | --- | --- | --- | --- | --- | --- | --- |
| **1.** | 38 | 2 | II/B | pT2b | pN1 | pM1 | 16 | Non keratinizing squamous cell carcinoma of cervix. Metastasis to regional lymph nodes. |
| **2.** | 53 | 2 | II/B | pT2b | pN1 | pM1 | 16 | Partly keratinizing squamous cell carcinoma of cervix. Metastasis to regional lymph nodes. |
| **3.** | 44 | 2 | I/B | pT1b | pN0 | pM0 | 16 | Partly keratinizing squamous cell carcinoma of cervix. |

Gr: Grade; F: FIGO stage; T: Tumor size; N: Node status; M: Metastasis
