# Supplementary material for: Two ways of epigenetic silencing of TFPI2 in cervical cancer
Source: PLoS One. 2020 Jun 19;15(6):e0234873. doi: 10.1371/journal.pone.0234873 (PMC7304613; doi:10.1371/journal.pone.0234873)
Supplement: S2 Table — (DOCX) [file pone.0234873.s003.docx]

**S2 Table. HPV type-specific nested PCR primer sequences.**

| **Primers** | **Orientation** | **Sequence (5’–3’)** |
| --- | --- | --- |
| Consensus outer | Forward | ACCGAAAACGGTTGAACCGAAAACGGT |
|  | Reverse | AATAATGTCTATATTCACTAATT |
| HPV16-specific | Forward | ATGTTTCAGGACCCACAGGA |
|  | Reverse | CCTCACGTCGCAGTAACTGT |
| HPV18-specific | Forward | ATGGCGCGCTTTGAGGATCC |
|  | Reverse | GCATGCGGTATACTGTCTCT |
| HPV33-specific | Forward | GCAGTAAGGTACTGCACCAC |
|  | Reverse | CCTCAGATCGTTGCAAAGGT |
