# Supplementary material for: Two ways of epigenetic silencing of TFPI2 in cervical cancer
Source: PLoS One. 2020 Jun 19;15(6):e0234873. doi: 10.1371/journal.pone.0234873 (PMC7304613; doi:10.1371/journal.pone.0234873)
Supplement: S3 Table — (DOCX) [file pone.0234873.s004.docx]

**S3 Table. Primer sequences to *TFPI2* HRM/pyrosequencing.**

| **Assay** | **Szekvencia (5’–3’ orientation)** | **Localization** | **Size (bp)** |
| --- | --- | --- | --- |
| ***4*** | F: GGGAGAGGAATTTTTAGTTAAGTTGAAAAGTTG | 20202-20234 | 203 |
|  | R^*^: ACCTTTCAAATACCTAAACTTCATACCT | 20377-20404 |  |
|  | S: TGTTTTTTAAATTTTTTTTTGTAGT | 20239-20263 |  |
| ***5*** | F^*^: GGTTAGATATTTGTTGGTTTTTGAGTAGTA | 19893-19922 | 324 |
|  | R: AAAAATTCCTCTCCCTCTTACACAATTTAC | 20187-20216 |  |
|  | S: AATCAACCACCCCTC | 20136-20150 |  |
| ***6*** | F: TTAGGTATTTGAAAGGTTGGTGGAGAGA | 20388-20415 | 214 |
|  | R^*^: CACAATTATCAAAATTTATCAAACTATCCACCTAC | 20567-20601 |  |
|  | S: TGGTGGAGAGAAAGT | 20405-20419 |  |

F: forward, R: reverse, S: sequencing primer; ^*^ biotinilated
