# Supplementary material for: Two ways of epigenetic silencing of TFPI2 in cervical cancer
Source: PLoS One. 2020 Jun 19;15(6):e0234873. doi: 10.1371/journal.pone.0234873 (PMC7304613; doi:10.1371/journal.pone.0234873)
Supplement: S4 Table — (DOCX) [file pone.0234873.s005.docx]

**S4 Table. Antibodies used.**

| **Antibody** | | **Host species, isotype** | **Manufacturer*** | **Cat. No.** | **Dilution IHC/IF** | **Dilution WB** |
| --- | --- | --- | --- | --- | --- | --- |
| **Primary** | Vimentin | Mouse monoclonal IgG, clone V9 | Dako | M0725 | 1:200 | - |
|  | Cytokeratin 8 | Rabbit polycolnal IgG | Abcam | AB52949 | 1:200 | - |
|  | Anti-TFPI-2 | Rabbit polycolnal IgG | Merck Millipore | ABT27 | - | 1:1,000 |
|  | Anti-TFPI-2 (PP5) | Rabbit polycolnal IgG | Dr. Hans Bohn | - | 1:500 |  |
|  | GAPDH | Mouse monoclonal IgG1, clone FF26A/F9 | AbD Serotec | MCA2427 | - | 1:2,000 |
|  | Beta-actin | Mouse monoclonal IgG, clone AC-74 | Sigma-Aldrich/Merck | A2228 | - | 1:4,000 |
| **Secondary** | Anti-mouse Ig/HRP | Goat polyclonal | DakoCytomation | P0447 | - | 1:2,000 |
|  | Anti-rabbit Ig/HRP | Goat polyclonal | DakoCytomation | P0448 | - | 1:2,000 |
|  | Anti-rabbit immunoglobulins/biotin | Rabbit polyclonal | DakoCytomation | E0447 | - | 1:1,000 |
|  | Alexa Fluor^®^ 488 anti-mouse IgG (H+L) | Donkey polyclonal | Invitrogen | A21202 | 1:200 | - |
|  | Alexa Fluor^®^ 568 anti-rabbit IgG (H+L) | Goat polyclonal | Invitrogen | A11011 | 1:200 | - |
| *Dako/DakoCytomation, Agilent Technologies, Inc., Santa Clara, CA. USA; Invitrogen by Life Technologies. Carlsbad. CA. USA; Merck Millipore Co., Darmstadt, Germany; Sigma-Aldrich, St. Louis, MO, USA; AbD Serotec, Kidlington, UK , Abcam, Cambridge, UK | | | | | | |

IHC: immunohistochemistry, IF: fluorescent cytochemistry, WB: Western blot
