# Supplementary material for: Two ways of epigenetic silencing of TFPI2 in cervical cancer
Source: PLoS One. 2020 Jun 19;15(6):e0234873. doi: 10.1371/journal.pone.0234873 (PMC7304613; doi:10.1371/journal.pone.0234873)
Supplement: S5 Table — (DOCX) [file pone.0234873.s006.docx]

**S5 Table.** **Data of *miR-23a* mimics and inhibitors from Exiqon.**

| **miR name (human)** | **Accession** | **Previous ID** | **microRNA target sequence** | **Cat. no. of** | |
| --- | --- | --- | --- | --- | --- |
|  |  |  |  | **Mimic** | **Power Inhibitor** |
| *hsa-miR-23a-3p* | MIMAT0000078 | *hsa-miR-23a* | 45-AUCACAUUGCCAGGGAUUUCC-65 | 470983-001 | 4103406-100 |
| *hsa-miR-23a-5p* | MIMAT0004496 | *hsa-miR-23a** | 9-GGGGUUCCUGGGGAUGGGAUUU-30 | 473453-001 | 4101840-101 |
