# Supplementary material for: Two ways of epigenetic silencing of TFPI2 in cervical cancer
Source: PLoS One. 2020 Jun 19;15(6):e0234873. doi: 10.1371/journal.pone.0234873 (PMC7304613; doi:10.1371/journal.pone.0234873)
Supplement: S6 Table — (DOCX) [file pone.0234873.s007.docx]

**S6 Table. Data of miRNA mimic and inhibitor negative controls from Exiqon.**

| **Name of Control miRNA** | **microRNA target sequence** | **Cat. no.** |
| --- | --- | --- |
| Negative Control 4 miRCURY LNA miRNA Mimic | GAUGGCAUUCGAUCAGUUCUA | 479903-001 |
| Negative control A, miRCURY LNA miRNA Inhibitor Control | TAACACGTCTATACGCCCA | 199006-100 |
| Negative control A, miRCURY LNA miRNA Inhibitor Control, 5’-fluorescein labeled | TAACACGTCTATACGCCCA | 199006-111 |
