# Supplementary material for: Two ways of epigenetic silencing of TFPI2 in cervical cancer
Source: PLoS One. 2020 Jun 19;15(6):e0234873. doi: 10.1371/journal.pone.0234873 (PMC7304613; doi:10.1371/journal.pone.0234873)
Supplement: S7 Table — (DOCX) [file pone.0234873.s008.docx]

**S7 Table**. **Clinico-pathological data of patients whose FFPE samples were used for IHC.**

| **Number of cases** | 31 | |
| --- | --- | --- |
| **Median age (years)** | 43 (29-71) | |
| **Histology** | *Number* | *%* |
| ***Adenosquamous carcinoma*** | 2 | 6.5 |
| ***Adenocarcinoma*** | 3 | 9.7 |
| ***Squamous cell carcinoma*** | 24 | 77.4 |
| ***Dysplasia*** | 1 | 3.2 |
| ***No tumor*** | 1 | 3.2 |
| **FIGO Stage** |  |  |
| ***IA*** | 4 | 12.9 |
| ***IB*** | 10 | 32.3 |
| ***IIA*** | 4 | 12.9 |
| ***IIB*** | 4 | 12.9 |
| ***No data*** | 9 | 29.0 |
| **Grade** |  |  |
| ***1*** | 3 | 9.7 |
| ***2*** | 13 | 41.9 |
| ***3*** | 2 | 6.5 |
| ***No data*** | 13 | 41.9 |
| **Metastasis** | 10 | 32.3 |
| **HPV status** |  |  |
| ***Only HPV16*** | 9 | 29.0 |
| ***Only HPV18*** | 1 | 3.2 |
| ***Only HPV33*** | 0 | 0.0 |
| ***HPV16+18*** | 2 | 6.5 |
| ***HPV16+33*** | 0 | 0.0 |
| ***HPV18+33*** | 0 | 0.0 |
| ***HPV16+18+33*** | 1 | 3.2 |
| ***HPV negative*** | 1 | 3.2 |
| ***No data*** | 17 | 54.8 |
