# Supplementary material for: Two ways of epigenetic silencing of TFPI2 in cervical cancer
Source: PLoS One. 2020 Jun 19;15(6):e0234873. doi: 10.1371/journal.pone.0234873 (PMC7304613; doi:10.1371/journal.pone.0234873)

Orginal gels and blots

Symbols:

NF: cells of normal fibroblast primary culture from case 2
MF: cells of metastatic-associted fibroblast primary culture from case 2
TF: cells of tumor-associted fibroblast primary culture from case 2
T: cells of tumor primary culture from case 2
C: cells of CSCC7 tumor cell line
NFT: NF+T direct co-culture
MFT: MF+T direct co-culture
TFT: TF+T direct co-culture
NFC: NF+C direct co-culture
MFC: MF+C direct co-culture
TFC: TF+C direct co-culture

MW: molecular weight
NC: negative control
1x, 10x, 100x: different dilutions
FFPE: formalin-fixed, paraffin-embedded tissue

M-NC: NF cells trasfected with mimic negative control
M-5p: NF cells trasfected with mimic *mir-23a-5p*
M-3p: NF cells trasfected with mimic *mir-23a-3p*
IH-NC: NF cells trasfected with inhibitor negative control
IH-5p: NF cells trasfected with inhibitor *mir-23a-5p*
IH-3p: NF cells trasfected with inhibitor *mir-23a-3p*
UT: untreated NF cells

X: lanes not included in the final figure

Original Western blots of Fig 3A, 3C, 3E.

Image capture: Ponceau/ECL and Kodak Image Station 4000MM (Eastman Kodak Company, Rochester, NY, USA).

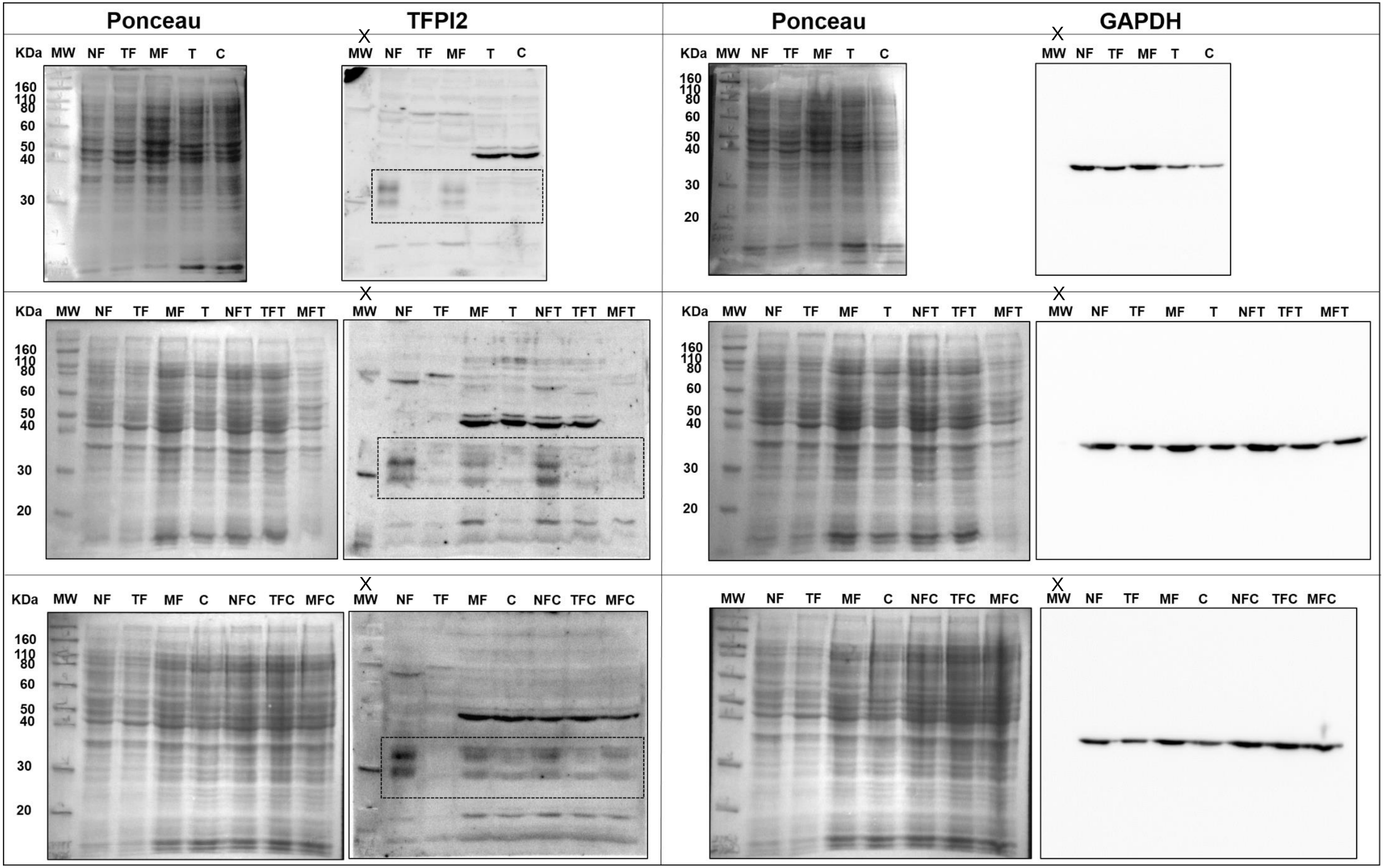

Original Western blots of Fig 5B.

Image capture: Ponceau/ECL and Kodak Image Station 4000MM (Eastman Kodak Company, Rochester, NY, USA).

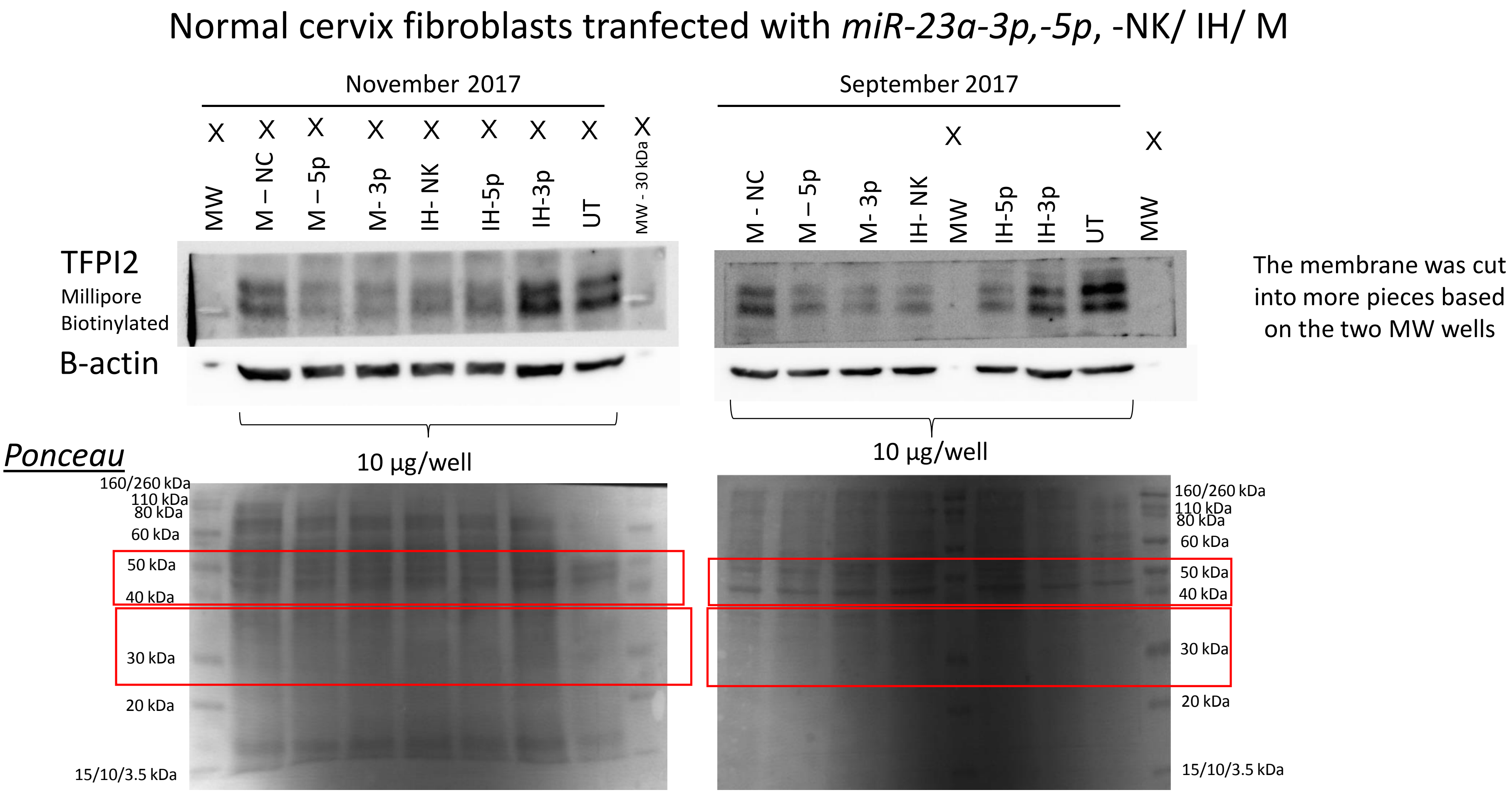

Original gels of Fig 7A.

Image capture: Kodak Image Station 4000MM (Eastman Kodak Company, Rochester, NY, USA).

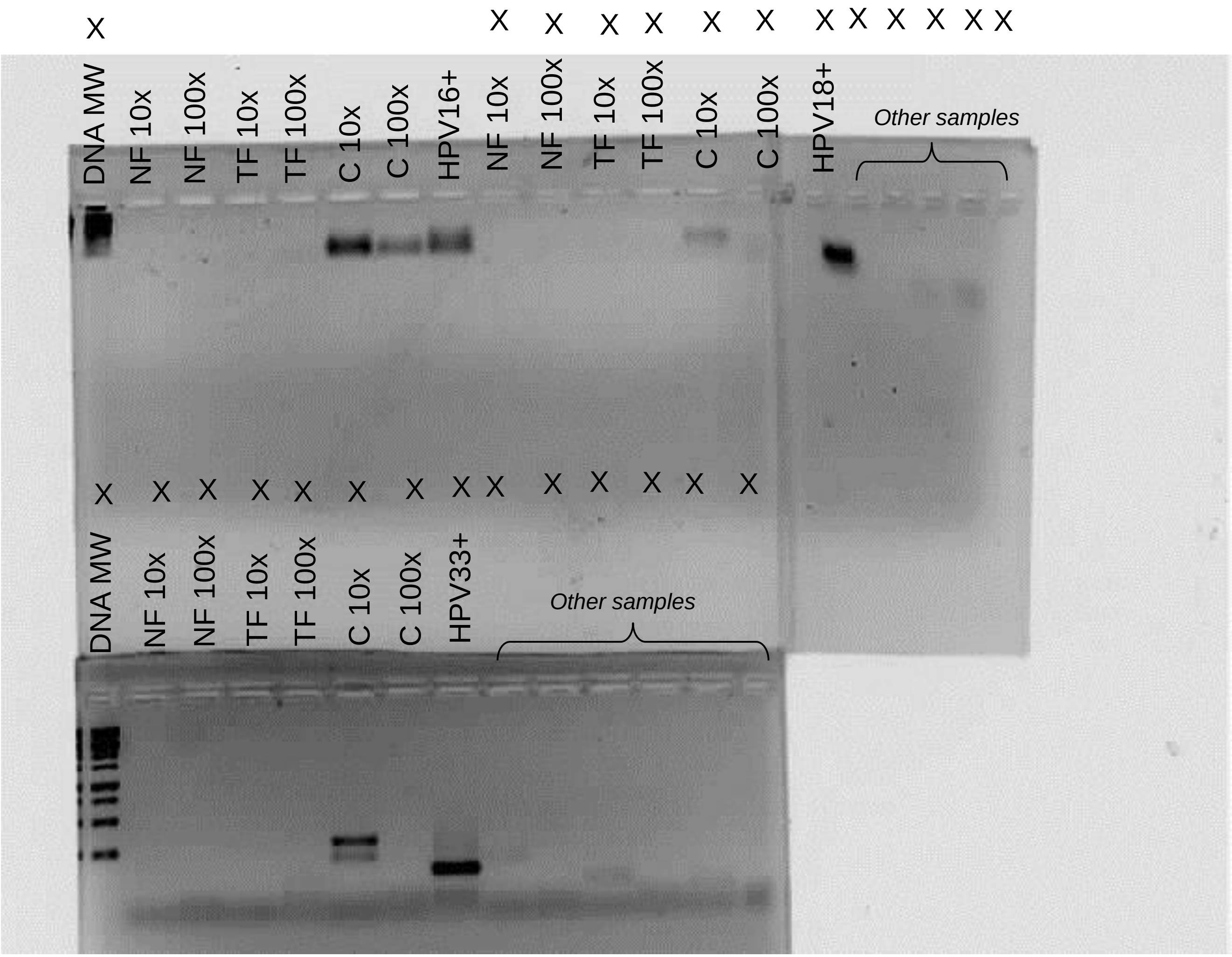

In the other wells other samples were which are not relevant to this study

Original gels of Fig 7B.

Image capture: iBright Imaging Systems (Thermo Fisher Scientific).

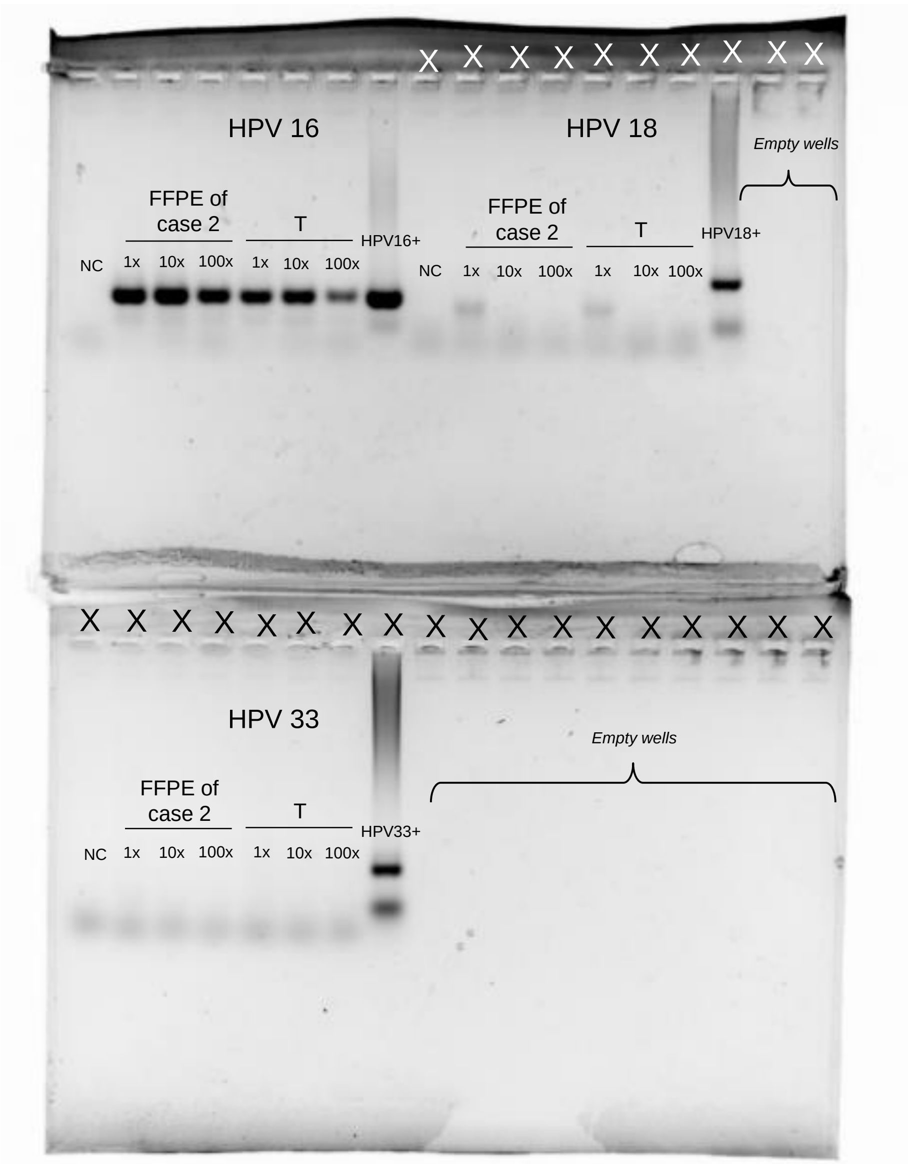

Supplement: S1 Raw Images — (PDF) [file pone.0234873.s009.pdf]
